# Protective effect of tolvaptan against cyclophosphamide‐induced nephrotoxicity in rat models

**DOI:** 10.1002/prp2.659

**Published:** 2020-09-30

**Authors:** Mohamed El‐Shabrawy, Amal Mishriki, Hisham Attia, Basma Emad Aboulhoda, Mohamed Emam, Hanaa Wanas

**Affiliations:** ^1^ Department of Medical Pharmacology Faculty of Medicine Cairo University Cairo Egypt; ^2^ Department of Toxicology and Pharmacology Faculty of Pharmacy Taibah University Madinah Saudi Arabia; ^3^ Department of Anatomy and Embryology Faculty of medicine Cairo University Cairo Egypt; ^4^ Department of Histopathology Faculty of Medicine Cairo University Cairo Egypt

**Keywords:** arginine vasopressin, cyclophosphamide, hyponatremia, inflammatory cytokines, nephrotoxicity, tolvaptan

## Abstract

Cyclophosphamide (CP) is a chemotherapeutic agent which is extensively used in the treatment of multiple neoplastic and nonneoplastic diseases like breast cancer, lymphomas, systemic lupus erythematosus, and multiple sclerosis. Dose‐limiting side effects, mainly nephrotoxicity is a major problem hindering its use in the clinical practice. CP induces nephrogenic syndrome of inappropriate antidiuresis mostly via the activation of arginine vasopressin V_2_ receptors. Moreover, CP produces reactive metabolites which is responsible for augmentation of lipid peroxidation and oxidative stress. Tolvaptan (TOL) is a selective vasopressin V_2_ receptor antagonist used in the treatment of clinically significant hyponatremia, volume overload in heart failure, and liver cirrhosis with edema. The present study aimed to investigate the potential protective effect of TOL in CP‐induced nephrotoxicity. Twenty‐four adult male albino rats were randomly divided into four groups: the control group, TOL group that treated daily with tolvaptan (10 mg/kg/d, orally), CP group where CP was administered intraperitoneally 75 mg/kg on days 3, 4, 5, 19, 20, and 21 of study, and the CP + TOL group where animals were treated with TOL daily with (10 mg/kg/d, orally) for 22 days with concomitant administration of CP as described before. Coadministration of TOL with CP induces significant improvement in the level of urine volume, serum Na+, serum osmolarity, urinary creatinine, and free water clearance in addition to significant reduction of body weight, serum creatinine, urea, serum K+, blood pressure, urine osmolarity, and the fractional excretion of sodium as compared to CP‐treated group. In addition, coadministration of TOL significantly reduced MDA, the marker of lipid peroxidation, and different pro‐inflammatory cytokines. Histopathological changes showed improvement in the signs of nephrotoxicity with the coadministration of TOL. Also, co‐treatment with TOL significantly decreased the level of markers of apoptosis as caspase‐3 and Bax with increased expression of antiapoptotic Bcl‐2 in renal tissue as compared to CP‐treated group.

## 1 INTRODUCTION

Since cyclophosphamide (CP) has been introduced into the clinical use in 1950s, it was considered to be one of the most potent chemotherapeutic agents. It is used widely to treat lymphomas and different solid malignancies in addition to various immunological diseases such as systemic lupus erythematosus and rheumatoid arthritis.^1^, ^2^ CP and its metabolites produce renal injuries involving renal cell apoptosis and fibrosis representing a major obstacle facing its usefulness.^3^ Protective agents against CP‐induced nephrotoxicity could offer the possibility of using higher doses of CP increasing cancer cure rates.

Hyponatremia is a serious metabolic disturbance which is commonly encountered in clinical practice in between cancer patients. Syndrome of inappropriate secretion of antidiuretic hormone (SIADH) is a common cause of hyponatremia in cancer patients. SIADH could be induced by some drugs including anxiolytics and narcotic drugs or may result from ectopic production of the antidiuretic hormone, arginine vasopressin (AVP) by the tumor tissue. Other factors can add to the possibility to develop significant hyponatremia including chemotherapy itself, treatment‐induced vomiting, and excessive hydration.^4^, ^5^, ^6^, ^7^

CP is converted in the liver into two reactive metabolites, acrolein and phosphoramide. They interfere with tissues' antioxidant capacity with subsequent production of highly reactive oxygen free radicals. These radicals induce considerable structural damage by interacting with proteins' amino acids and DNA, producing irreversible DNA cross‐links and leads to cell apoptosis.^8^, ^9^, ^10^, ^11^, ^12^, ^13^

One of the main mechanisms by which CP produces hyponatremia is drug‐induced nephrogenic syndrome of inappropriate antidiuresis (NSIAD). CP activates the antidiuretic hormone arginine vasopressin (AVP) receptors V_2_ with subsequent upregulation of the water channels, aquaporin_2_ (AQP_2_), in kidney without vasopressin stimulation.^11^, ^14^

The AVP receptor antagonists as tolvaptan (TOL) correct hyponatremia by directly blocking the binding of AVP with its receptors. In clinical trials, TOL has increased serum osmolality and normalized the serum Na^+^ in hyponatremia associated with SIADH, cirrhosis, or congestive heart failure. These drugs may have a potential role in cancer‐related hyponatremia as well.^15^

Conventional treatments of hyponatremia, such as fluid restriction, hypertonic saline, demeclocycline, and diuretics have not been well tolerated due to associated electrolyte abnormalities, neurohormonal activation, renal dysfunction, and possible increased mortality.^16^ As traditional treatments for hyponatremia are difficult for patients to tolerate, have marginal efficacy, and could result in electrolyte imbalances, vasopressin antagonists were developed specifically to counteract the abnormal pathophysiology resulting from an excess of AVP.^17^

Noteworthy, TOL has been recently shown to be significantly nephroprotective in a rat cirrhotic model ^18^ where TOL increases water diuresis leading to an increase in intravascular sodium level and migration of extravascular fluid into blood vessels. The ability of TOL to preserve the intravascular fluid adds an advantage to its diuretic effect in the form of proper maintenance of the arterial blood pressure. So it can potentially increase the renal blood flow and protect the kidney.^19^ This study aimed to investigate the potential protective effect of TOL in CP‐induced nephrotoxicity.

## 2 MATERIALS AND METHODS

### 2.1 Drugs

Cyclophosphamide monohydrate (218707) and tolvaptan (T7455) were obtained from Sigma‐Aldrich and administered as a freshly prepared solution in saline.

### 2.2 Animals and experimental design

Adult male albino rats, each weighing 200‐220 g, were kept under standard conditions (25 ± 2°C room temperature; 12‐hour light/dark cycle with lights on at 07:30 AM), with free access to standard pelleted diet and water ad libitum. All the animal experiments were approved by the Institutional Animal Care and Use Committee of Cairo University, and were implemented in strict accordance with approved guidelines. All the animals were randomly divided into one of the four groups, six rats in each group as follows:

#### 2.2.1 G‐l, control group

Animals were injected with normal saline (2.5 ml/kg) intraperitoneally (i.p.) on days 3, 4, 5, 19, 20, and 21. They were given normal saline orally from day 1 to day 22.

#### 2.2.2 G‐ll, TOL group

Animals were treated daily with tolvaptan (10 mg/kg/d, orally) for 22 days.^18^ Animals were injected with normal saline (2.5 ml/kg) intraperitoneally (i.p.) on days 3, 4, 5, 19, 20, and 21.

#### 2.2.3 G‐lll, CP group

Animals were injected i.p. with CP (75 mg/kg) on days 3, 4, 5, 19, 20, and 21. And they were given normal saline orally from day 1 to day 22.^20^

#### 2.2.4 G‐lV, CP + TOL group

Animals were treated with TOL daily with (10 mg/kg/d, orally) for 22 days. Concomitantly animals were injected i.p. with CP (75 mg/kg) on days 3, 4, 5, 19, 20, and 21.^18^, ^20^

Urine was collected at day 22 in individual metabolic cages. Urine volume was assessed and then urine was centrifuged and clear supernatant was kept at −20°C for further investigation. After receiving the last dose of drugs, body weight was measured as a measure of toxicity and systolic blood pressure was assessed using a tail‐cuff blood pressure measuring system as described below. At the end of the experiment, all rats were sacrificed and blood samples were collected and centrifuged and serum samples stored at −20°C for further investigation. The longitudinal section of the right kidney from each animal was used for histological examination. The rest of kidneys were frozen at −80°C for molecular analysis.

### 2.3 Assessment of arterial blood pressure

It was done by using a tail‐cuff blood pressure measuring system (Harvard Apparatus Ltd, Edenbridge, Kent, England). In the tail‐cuff technique, animals were warmed for 30 minutes at 28°C in a thermostatically controlled heating cabinet (UgoBasille, Italy) for better detection of tail artery pulse, the tail was passed through a miniaturized cuff and a tail‐cuff sensor that was connected to an amplifier (ML 125 NIBP, AD Instruments, Australia).^21^ Systolic blood pressure (SBP) was measured five consecutive times in conscious rats by a tail‐cuff plethysmograph after the animals had rested for at least 15 minutes; the mean value of the lowest three readings was recorded.

### 2.4 Assay of kidney function markers

Commercially available kits supplied by the Egyptian company for biotechnology, Bio diagnostic, CO, Egypt were used to measure serum creatinine, serum urea, blood urea nitrogen (BUN), blood glucose, serum Na^+^, K^+^ following the manufacturer's instructions.

Plasma osmolality (**P**_osm_) was estimated by using the following equation^22^

$$\begin{array}{c} \text{Plasma osmolality}‐2{\text{Na}}^{+}+\left(\text{BUN}÷2.8\right)+\left(\text{glucose}÷18\right)\\ \text{BUN}:\text{blood urea nitrogen}\;\text{Na}+:\text{sodium} \end{array}$$

Na^+^ and creatinine in urine samples were measured using an automatic biochemistry analyzer (CLINITEK Status, Siemens). Urine osmolarity (**U**_osm_) was measured using osmometer (Osmette A).

(**U**_osm_), urinary creatinine and urinary Na^+^ for calculation of fractional excretion of sodium (**FeNa**), and free water clearance (**C_H2o_**) were measured using the following equations^23^, ^24^:

$$C_{H_{2}O}=V‐\frac{U_{\text{osm}}}{P_{\text{osm}}}V=\left(1‐\frac{U_{\text{osm}}}{P_{\text{osm}}}\right)V,$$

$${\text{FE}}_{\text{Na}}=100\times \frac{{\text{sodium}}_{\text{urinary}}\times {\text{creatinine}}_{\text{plasma}}}{{\text{sodium}}_{\text{plasma}}\times {\text{creatinine}}_{\text{urinary}}},$$

### 2.5 Histopathological examination

The other kidney was also removed and kept in 10% phosphate‐buffered formalin. Samples were fixed and embedded in paraffin wax, and 3‐ to 5‐μm thick sections were stained for histopathological diagnosis with hematoxylin and eosin (H & E staining),^25^ Periodic Acid‐Schiff (PAS)^26^ was used to evaluate any thickening of basement membranes and any sclerotic parameters, and Masson's trichrome^27^ staining for the assessment of any interstitial fibrosis.

### 2.6 Measurement of MDA

Animals were sacrificed. One kidney was immersed in liquid nitrogen, then it was kept at −85°C for colorimetrical determination of renal malondialdehyde (MDA) content, a product of lipid peroxidation. This compound is a reactive aldehyde and is one of the many reactive electrophile species that cause toxic stress in cells and form covalent protein adducts referred to as advanced lipoxidation end products, in analogy to advanced glycation end products. The production of this aldehyde is used as a biomarker to estimate the level of oxidative stress in kidney as mentioned by Ref. [28]

### 2.7 Assay of pro‐inflammatory cytokines in renal tissue

TNF‐α, NF‐κB, IL‐1β, and IL‐6 levels were determined in the renal tissue by using rat enzyme‐linked immunosorbent assay (ELISA) kit which were obtained from Bio‐diagnostic Company, Egypt. Analysis was performed by Elisa Plate Reader (Bio‐Tek).

### 2.8 Immunohistochemistry

Tissue sections were de‐paraffinized and rehydrated using a graded ethanol series.

After antigen retrieval, eliminating endogenous peroxidase and preincubating with 5% BSA to block background staining, sections were incubated with mouse polyclonal anti‐Bax antibody (1:100) (ab216494), rabbit polyclonal anti‐Bcl‐2 antibody (1:50) (ab196495), and rabbit polyclonal anti‐cleaved caspase‐3 antibody (ab2302). All antibodies were purchased from Abcam^®^ (Cambridge). The color reaction was then made with HRP‐linked polymer detection system and counterstained with hematoxylin as previously described.^29^ Paraffin‐embedded mouse spleen tissue was used as a positive control. Negative controls were processed via omission of the primary antibodies in the automated staining protocol.

### 2.9 Histomorphometric study

A quantitative study was performed using Leica Qwin 500 image analyzer computer system (England) to assess the mean area percent of collagen fibers deposition in Masson's trichrome stained sections as well as the area percent of immunohistochemically positive structures. The image analyzer consisted of a colored video camera, colored monitor, hard disk of IBM personal computer connected to the microscope and controlled by Leica Qwin 500 software.

The image analyzer was first calibrated automatically to convert the measurement units (pixels) produced by image analyzer program into actual measurement units. Digital images were uniformly acquired and snapped at x400 magnification by Olympus IX51 equipped with a DP72 device camera. The obtained data were calculated in six nonoverlapping fields per slide in each group.

### 2.10 Statistical analysis

SPSS version 23.0 was used in data management. Data were tested for normality to select the appropriate test and data descriptors. Mean and standard deviation were used for data description. Parametric and nonparametric ANOVA were used to test differences for parameters measured at day 22 and inflammatory markers between study groups. Pairwise comparisons were Bonferroni adjusted. Correlation analysis was used to test for strength of association of inflammatory markers and other parameters at day 22. Scatterplots were drawn to show pattern and direction of association. P value was always two tailed and significant at 0.05 level.

## 3 RESULTS

### 3.1 Effect on body weight and systolic blood pressure

The cumulative dose of CP caused statistically significant increase in mean body weight and SBP (*P* < .05) on day 22 as compared to control group. Coadministration of TOL with CP produced statistically significant decrease in the mean body weight and SBP on day 22 compared with CP group (*P* < .05) as shown in Table 1.

**TABLE 1:** Body weight and systolic blood pressure at day 22

Parameter	Control	TOL	CP	CP + TOL
Body weight (g)	220.625 ± 1.7678^a^	221.875 ± 3.7201^a^	238.125 ± 2.5877^b^	223.125 ± 2.5877^a^
SBP (mmHg)	127.5000 ± 7.07107^a^	118.7500 ± 9.91031^a^	161.2500 ± 8.34523^b^	126.2500 ± 7.44024^a^

Note

Different superscripts (a–c) in the same row indicate significant difference (*P* < .05) among groups

### 3.2 Effects of tolvaptan on renal functions

Table 2 shows blood and urine data obtained on the final day of the animal experiment compared with vehicle‐treated control. It was found that there was significantly elevated serum levels of urea, creatinine, and K^+^ (*P* < .05) in CP‐treated group as compared with the control group. Coadministration of TOL significantly reduced the levels of urea, creatinine, and K^+^ as compared to the CP‐treated group (*P* < .05). CP treatment caused statistically significant decline in the plasma Na^+^ and serum osmolarity as compared to control group. The coadministration of TOL and CP showed statistically significant decline in the plasma Na^+^ and serum osmolarity as compared to CP group.

**TABLE 2:** Blood and urine data at day 22

Parameter	Control	TOL	CP	CP + TOL
Urea (mg/dL)	39.500 ± 1.0690^a^	41.250 ± 4.4320^a^	67.625 ± 1.8468^b^	49.750 ± 1.1650^c^
Creatinine (mg/dL)	0.4125 ± 0.27999^a^	0.2625 ± 0.10607^a^	2 ± 0.16903^b^	0.7125 ± 0.08345^c^
Serum Na^+^ (mmol/L)	147.250 ± 5.2576^a^	149.875 ± 2.6959^a^	118.625 ± 1.3025^b^	134.000 ± 1.7728^c^
Serum K^+^ (mmol/L)	4.038 ± 0.2264^a^	4.375 ± 0.6112^a^	5.713 ± 0.0991^b^	4.238 ± 0.2134^a^
Serum osmolarity (mosmole/kg H_2_O)	301.0913 ± 10.39010^a^	305.9050 ± 5.29577^a^	245.1225 ± 2.72161^b^	274.5975 ± 2.84485^c^
Urine volume (mL)	9.75 ± 0.267^a^	11.88 ± 0.354^b^	5.94 ± 0.320^c^	8.25 ± 0. 267^d^
Urinary creatinine (mg/dL)	71.250 ± 4.0620^a^	70.000 ± 6.5465^a^	49.875 ± 1.2464^b^	63.750 ± 2.2520^c^
Urinary Na^+^ (mEq/L)	92.025 ± 1.4109^a^	91.750 ± 1.8323^a^	93.375 ± 1.7678^a^	92.875 ± 1.8077^a^
FeNa	0.3587 ± 0.23955^a^	0.2338 ± 0.10378^a^	3.1563 ± 024242^b^	0.7763 ± 0.09870^c^
C_H2O_	−0.004225 ± 0.0008345^a^	−0.005700 ± 0.0006047^b^	−0.010362 ± 0.0006435^c^	−0.007813 ± 0.0002850^d^
Urine osmolarity (mosmole/kg H_2_O)	565.000 ± 43.6447^a^	516.250 ± 20.6588^b^	861.250 ± 33.9905^c^	650.000 ± 15.1186^d^

Note

Different superscripts (a‐c) in the same row indicate significant difference (*P* < .05) among groups.

TOL group was performed to show the effect of TOL on water and electrolyte balance with normal kidney in comparison to the control group: as shown TOL performed its effect by regulating the free water excretion that resulted in decreasing the urine osmolarity.

CP treatment caused statistically significant decline (*P* < .05) in 24 hours urinary volume, urinary creatinine, and free water excretion (C_H2O_) as compared to control group. Coadministration of TOL with CP resulted in significant increase (*P* < .05) in the three parameters as compared to CP group.

In contrast, CP and CP + TOL groups did not show statistically significant change in urinary sodium as compared to control group.

Urinary osmolarity and FeNa were significantly increased (*P* < .05) in CP group in comparison to control group. CP + TOL group showed significant decrease of both parameters when compared with CP group.

### 3.3 Effects of tolvaptan on renal histological changes

**FIGURE 1:**
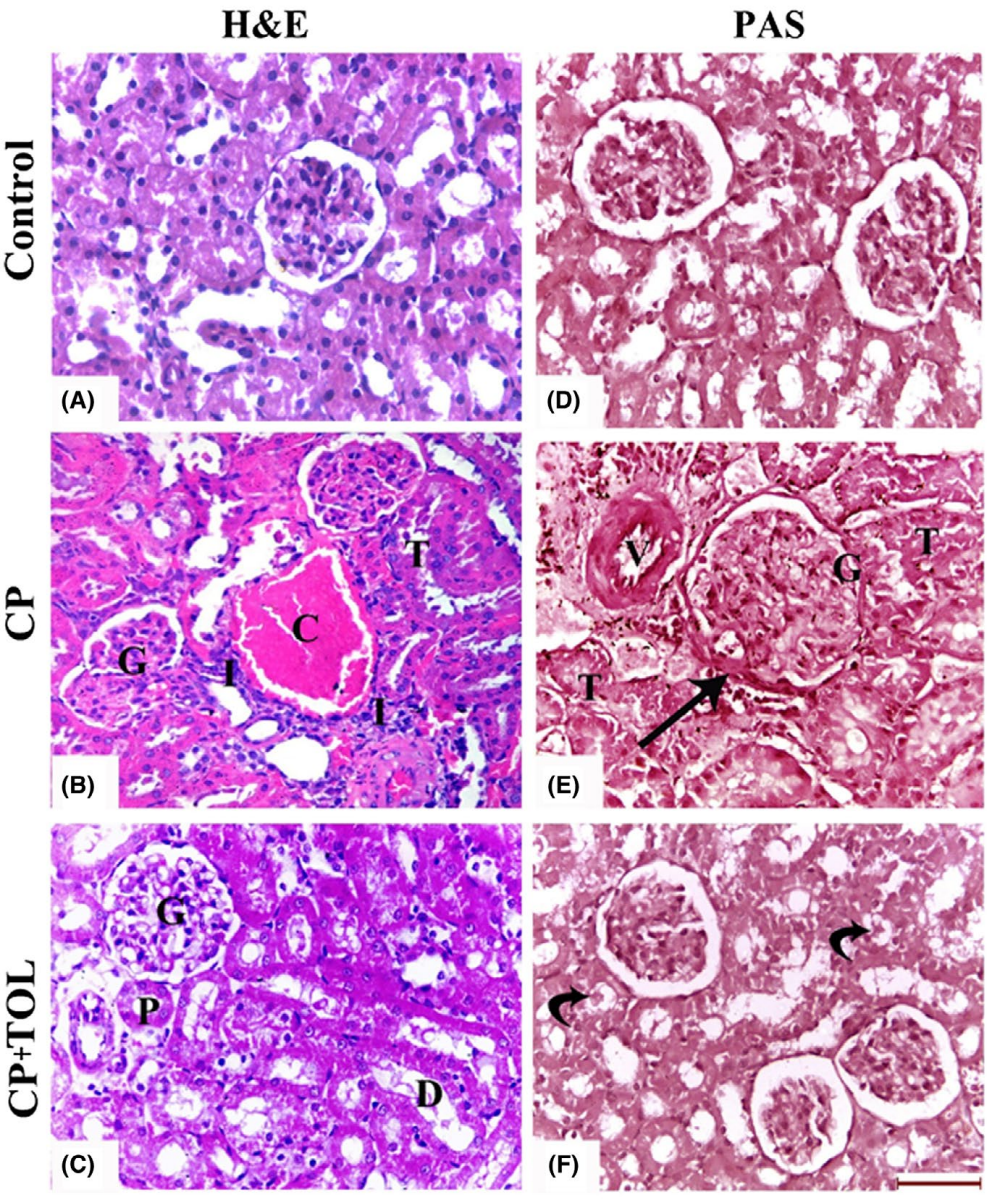
H&E‐stained sections of (A): Control group, (B) CP‐treated group showing marked congestion (C) of the blood vessels and inflammatory cellular infiltration (I). Note: distortion of the tubules (T) and glomeruli (G), (C) CP + TOL‐treated group showing improvement in the structural pattern of the glomeruli (G), proximal (P), and distal (D) convoluted tubules, Periodic Acid‐Schiff (PAS)‐stained sections of (D): Control group, (E) CP‐treated group showing thickening of the Bowman's capsule (arrow) and wall of blood vessels (V). The renal tubules (T) show disruption of their basal laminae and appear obliterated with casts. (F), CP + TOL‐treated group showing normal glomerular PAS reaction. The tubules exhibit few casts (curved arrows; (Scale bar 50 μm)

By H&E staining (Figure 1A‐C), sections from CP group showed marked congestion and infiltration by inflammatory cells in addition to tubular and glomerular distortion. On the other hand, TOL co‐treatment showed improvement in the structural pattern of the glomeruli and tubules as compared to CP group.

By PAS staining (Figure 1D‐F), sections from CP group showed thickening of the Bowman's capsule and wall of blood vessels in addition to the basal lamina of the renal tubules. On the other hand, sections from CP +TOL group showed normal reaction with PAS stain as compared to CP group.

**FIGURE 2:**
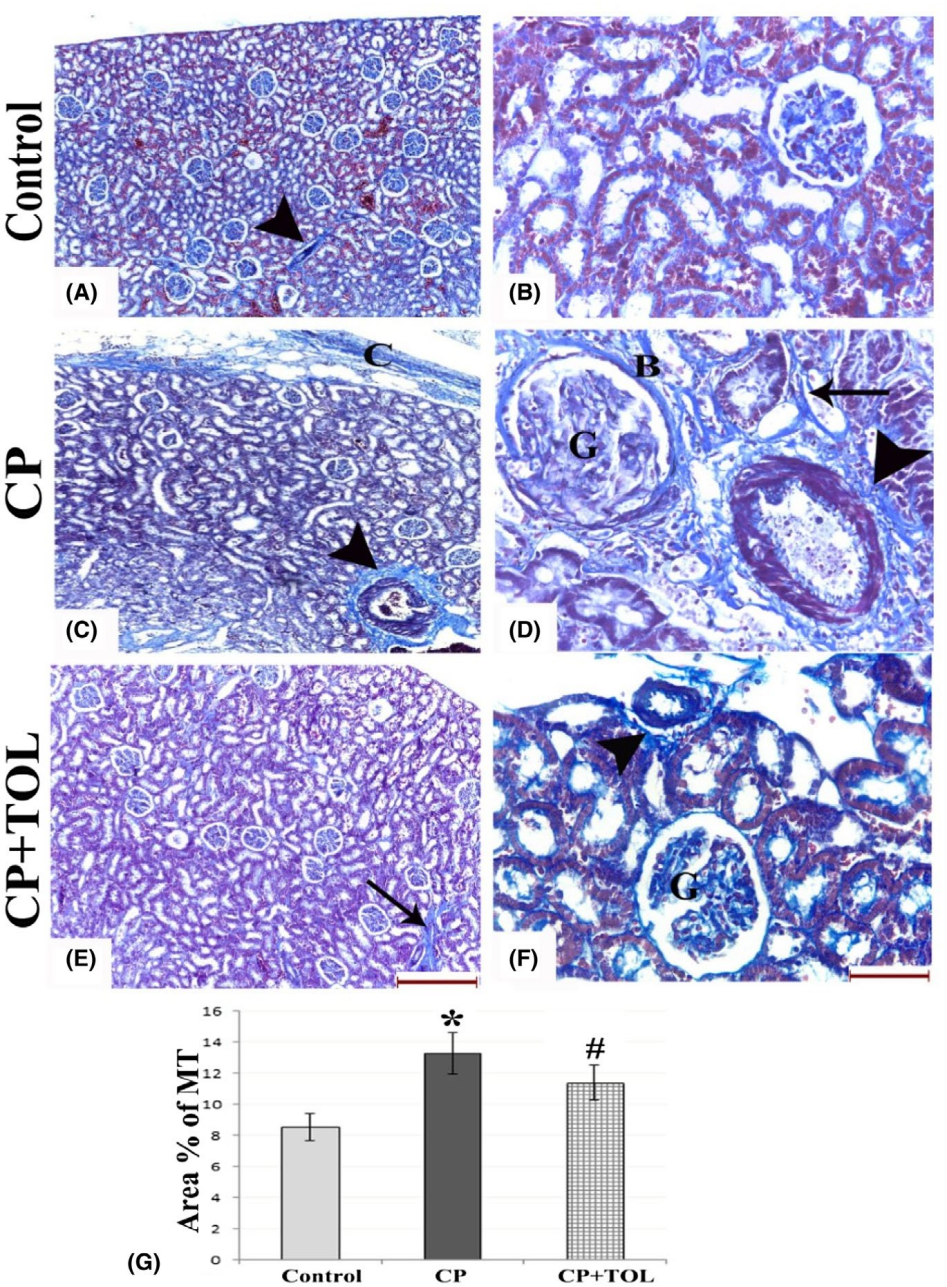
MT‐stained sections of (A,B) control group showing normal glomerular, interstitial, and perivascular (arrow head) collagen, (C,D) CP‐treated group showing increased capsular thickening (C) and dens deposition of collagen bundles around the blood vessels (arrow heads), around the tubules (arrow), in the Bowman's capsules (B), and within the glomeruli (G) with capillary to capsule adhesion. (E,F) CP + TOL‐treated group showing few collagen fibers within the glomeruli (G), around blood vessels (arrow head), and tubules (arrow). (G) Graph showing area percent of collagen deposition in different groups; data are presented as mean ± standard deviation, *: statistically significant relative to control group, #: statistically significant relative to CP group at *P* > .05 using ANOVA, Bonferroni post hoc pairwise comparison (n = 3; (a,c,e: scale bar 200 µm, b,d,f: scale bar 50 µm)

By MT staining (Figure 2), sections from CP group showed marked fibrosis and collagen deposition. CP +TOL‐treated group showed significant decrease in the area percent of collagen deposition when compared to CP group (P < .05).

**FIGURE 3:**
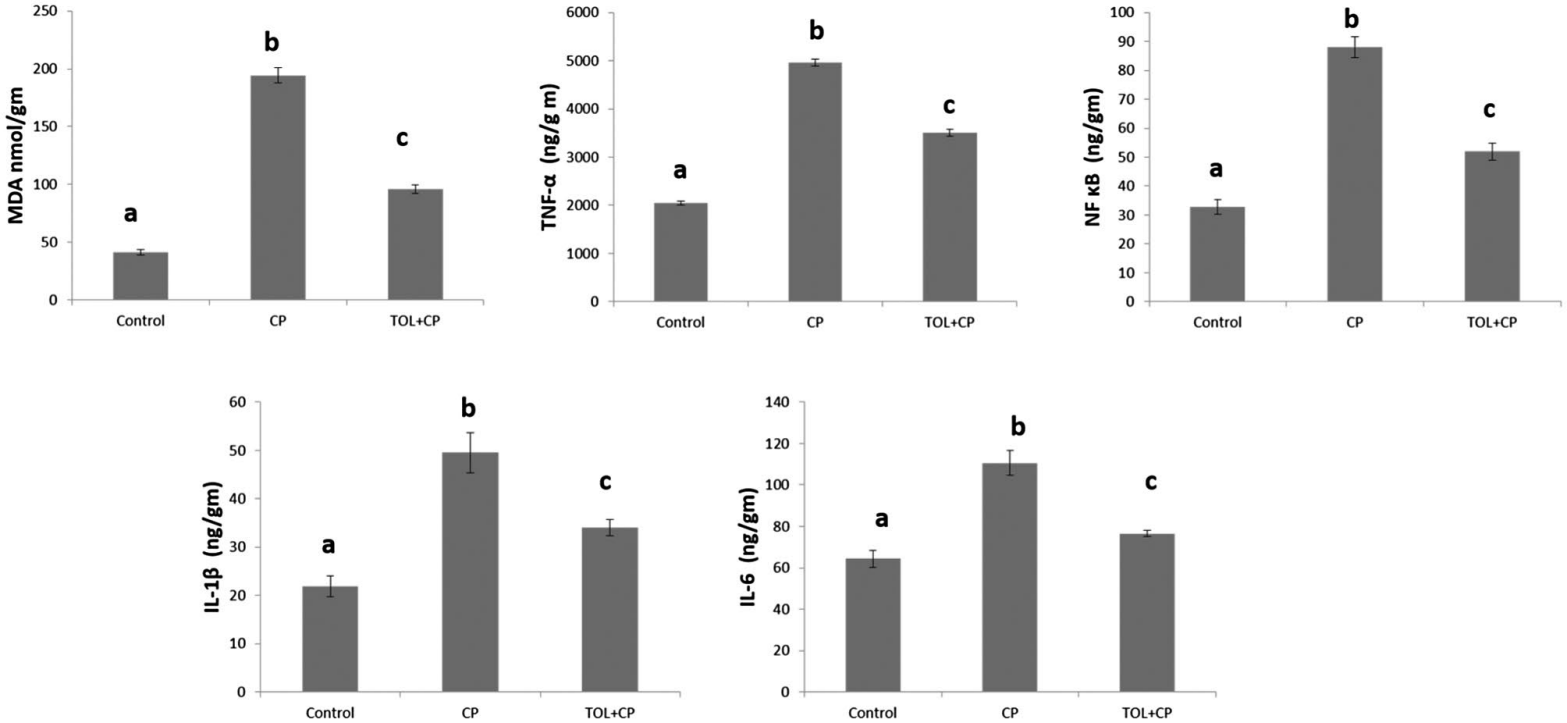
Effects of tolvaptan on malondialdehyde (MDA) and the pro‐inflammatory cytokines in renal tissues of CP‐induced nephrotoxicity in rats: (A‐C) Different superscripts in the same graph show a statistical difference (*P* < .05). Data represent the means ± SEM of six rats in each group

### 3.4 Effects of tolvaptan on renal malondialdehyde

Oxidative stress was evaluated through measuring renal MDA. Renal MDA was assessed as an index of renal lipid peroxidation. It was found that there was significantly elevated renal MDA in CP‐treated group as compared with control group (*P* < .05). However, TOL treatment in CP + TOL group significantly suppressed lipid peroxidation (*P* < .05) as compared with the CP‐treated group (Figure 3).

### 3.5 Effects of tolvaptan on pro‐inflammatory cytokines

CP treatment induced the pro‐inflammatory markers and induced significant increase (*P* < .05) of TNF‐α, NF κB, IL‐1β, and IL‐6 in comparison to control group. While co‐treatment with TOL showed significant decline (*P* < .05) of the four markers in comparison to the CP group (Figure 3).

### 3.6 Immunohistochemical findings of caspase‐3, BAX, and BCL‐2

**FIGURE 4:**
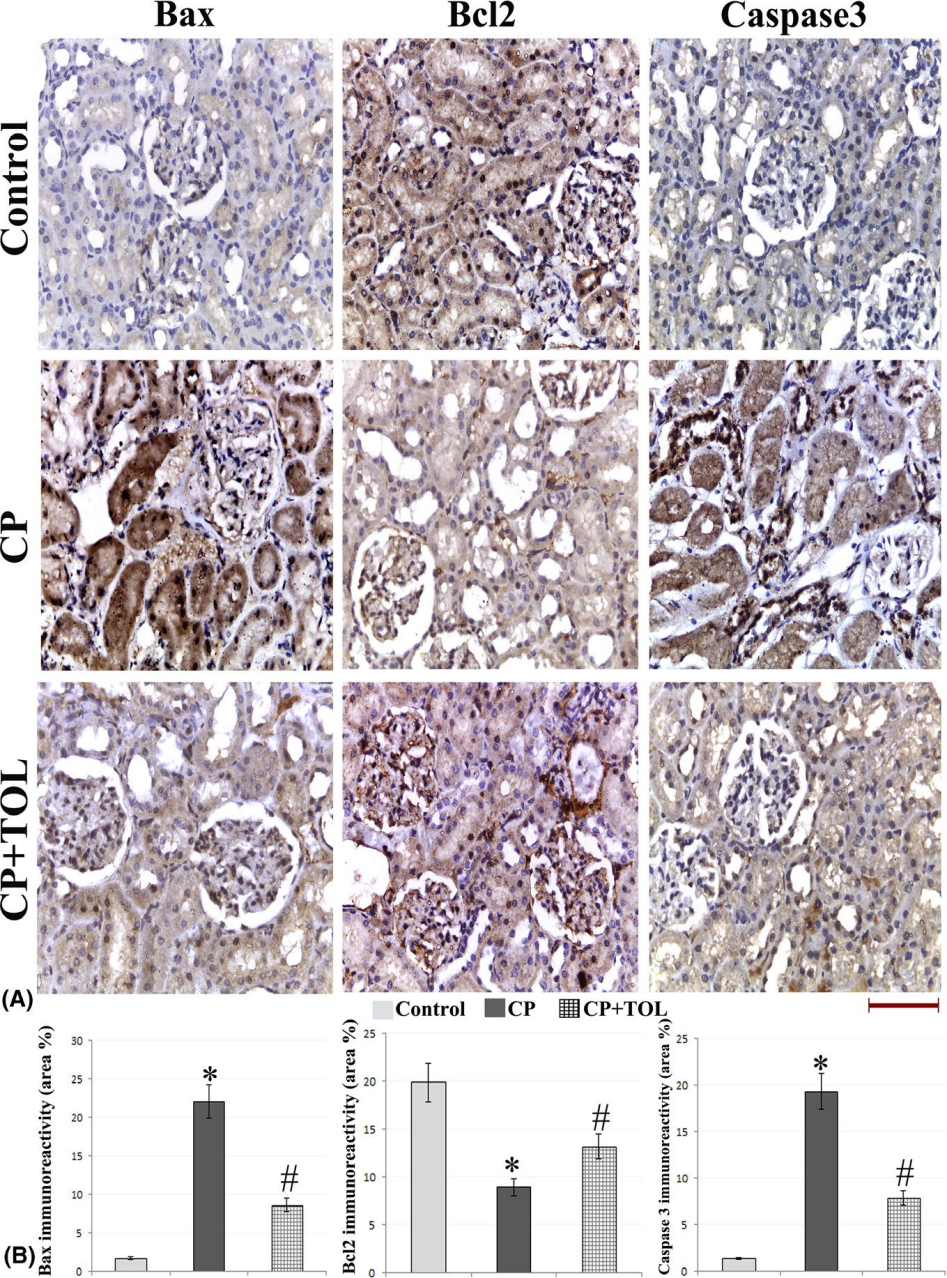
(A) Representative figures of Bax, Bcl‐2, and caspase‐3 immunohistochemical expression in the different study groups (g) Area percent of immunoreactivity of Bax, Bcl‐2, and caspase‐3 (n = 3); data are presented as mean ± standard deviation, *: statistically significant relative to control group, #: statistically significant relative to CP group at *P* > .05 using ANOVA, Bonferroni post hoc pairwise comparison (scale bar 50 µm)

When kidney tissues were examined immunohistochemically (Figure 4), CP‐treated group showed significant increase in caspase‐3 and Bax expressions in comparison to the control group (*P* < .05). TOL coadministration significantly reduced these expressions when compared to rats treated with CP only (*P* < .05).

In contrast, immunohistochemical analysis of Bcl‐2 staining showed significant reduction of Bcl‐2‐positive cells in CP‐treated rats as compared to the control group (*P* < .05). Concomitant administration of TOL resulted in a significant (*P* < .05) increase in the number of Bcl‐2‐positive cells when compared to CP group.

## 4 DISCUSSION

CP is a chemotherapeutic agent which is extensively used in the treatment of multiple malignancies and immunological diseases.^30^ Severe hyponatremia is a serious, potentially life‐threatening condition which is commonly encountered in clinical practice with the use of CP high doses. However, in some situations the condition also was reported with low doses of CP and led to severe acute hyponatremic encephalopathy.^31^ Additionally, dose‐related CP‐induced nephrotoxicity is one of the main causes of severe morbidity in surviving cancer patients.^32^

In this study, CP treatment demonstrated significant nephrotoxic effects, as confirmed by the elevation of urea and creatinine in addition to the increase in the serum K^+^ level and the decrease in the urinary creatinine excretion. These findings are supported by a previous study that demonstrated reduced elimination of K^+^ and increased plasma levels of creatinine and urea following a single dose of CP.^33^

Moreover, histopathological changes indicated nephrotoxicity induced by the cumulative dose of CP. The CP‐treated group showed increased capsular thickening and dens deposition of collagen bundles around the blood vessels, around the tubules, in the Bowman's capsules, and within the glomeruli in addition to capillary to capsule adhesion. The histopathological finding of this study is in agreement with Abraham and Isaac, 2011 who demonstrated similar changes in renal tissue due to CP toxicity.^34^

Coadministration of TOL with CP provided nephroprotective effect appeared as significant reduction in serum urea, creatinine, serum K^+^ and significant increase of the urinary excretion of creatinine when compared to group treated with only CP. In addition, histopathological changes with TOL co‐treatment indicate improvement in the signs of nephrotoxicity where there was few collagen fibers within the glomeruli, around blood vessels and tubules in comparison to CP group. Our study is in agreement with a previous study which demonstrated the renal decongestion and renoprotective effects of TOL in hypertensive heart failure rat model treated by TOL. They showed that renal fibrosis was significantly inhibited in the TOL‐treated groups compared to the control group. Authors showed that the % area fibrosis was significantly correlated with renal function and hemodynamic and ultrasound parameters of the kidney. These findings suggest a close relationship between renal congestion and renal fibrosis in addition to the close relationship between renal function and renal fibrosis.^35^

In this study, treating rats with cumulative dose of CP resulted in statistically significant decrease in urine volume, serum Na^+^, serum osmolarity, and the free water clearance in addition to significant increase in body weight, blood pressure, urine osmolarity, and the fractional excretion of sodium as compared to control group.

CP induces a state of nephrogenic syndrome of inappropriate antidiuresis with subsequent fluid retention and hyponatremia. CP has the ability to activate the AVP receptors V_2_ by upregulating AQP_2_ in kidney without AVP stimulation.^11^, ^14^

In our results, the marked reduction in fractional sodium excretion in the CP‐treated rats reflects the decreased ability of the kidneys to retain sodium due to the intrinsic renal impairment. Also our results showed that, excessive water accumulation was noticed in CP‐treated rats appeared in lowering the free water clearance with subsequent dilutional hyponatremia. Accumulation of water usually arises because of reduced renal excretion of water rather than excessive water intake as kidneys normally have a great capacity for water excretion.

In agreement with our results, a previous study showed 31% reduction in serum sodium concentration in rats treated with CP.^36^ Another study showed that CP decreases free water clearance and induces water retention by the water channels.^37^

The finding of the present study is contradictory to Omole et al, 2018 who determined a decrease in body weight of rats in CP‐induced cardiotoxicity in rat model.^38^ However, we can explain the increase in the body weight in our results by the decline of C_H2O_. Fluid retention resulted from decreased free water clearance can explain the increased body weight in CP group. Reduced urine volume, hyponatremia, diminished serum osmolarity, and elevated urine osmolarity in our results also are going with the decline in C_H2O_ and fluid retention.

Our results showed that coadministration of TOL with CP produced statistically significant increase in urine volume, serum Na^+^, serum osmolarity, and C_H2O_ in addition to significant decrease in body weight, blood pressure, urine osmolarity, FeNa in comparison to rats treated with CP only.

The findings of the present study are going with the results of Nakanishi, et al, 2016 who showed that TOL reduced urine osmolarity in patients with liver cirrhosis.^39^ Also, the role of TOL in the treatment of acute hyponatremia associated with acute kidney injury was shown in the results of Gopinath, et al, 2013.^40^

This study is in agreement with a previous study where TOL significantly reduced the SBP in the rat model of heart failure.^35^

Tanabe, et al., 2017, using rat model of cirrhosis, showed that TOL protected the kidney via increasing water diuresis leading to an increase in intravascular sodium level and movement of extravascular fluid into blood vessels with subsequent increase in the renal blood flow.^18^

The results of Morooka, et al., 2012 were in agreement with our study which showed that administration of oral TOL offers protection to the heart and kidneys in the rat model of with hypertensive heart failure and increase daily urine output with decrease serum creatinine level.^41^

Hyponatremia is a serious electrolyte disorder that occurs frequently in cancer patients. SIADH, either drug induced or due to ectopic hormone secretion by the cancer cells, represents a common cause of hyponatremia in such patients. However, there are other factors that can add to the risk to develop hyponatremia including chemotherapy itself, chemotherapy‐induced vomiting, and excessive hydration.^4^, ^5^, ^6^, ^7^, ^31^

Conventional treatments of hyponatremia, such as fluid restriction, hypertonic saline, demeclocycline, and diuretics, have not been well tolerated due to associated electrolyte abnormalities, neurohormonal activation, renal dysfunction, and possible increased mortality.^16^

As traditional treatments for hyponatremia are difficult for patients to tolerate, have marginal efficacy, and could result in electrolyte imbalances, vasopressin antagonists were developed specifically to counteract the abnormal pathophysiology resulting from an excess of AVP.^17^

The AVP receptor antagonists, such as conivaptan, TOL, and satavaptan, correct hyponatremia by directly blocking the binding of AVP with its receptors. In clinical trials, conivaptan, TOL, and satavaptan have increased serum osmolality and normalized the serum Na^+^ in hyponatremia associated with SIADH, cirrhosis, or congestive heart failure. These drugs may have a potential role in cancer‐related hyponatremia as well.^18^

TOL is a selective vasopressin V_2_ receptor blocker which is clinically used in the treatment of significant hyponatremia which can associate volume overload in heart failure and liver cirrhosis with edema. TOL, by blocking V2 receptors in the collecting duct cells, causes dislocation of AQP_2_ from the apical membrane to the cytoplasm with subsequent inhibition of water reabsorption and enhancement of urine excretion^15^, ^42^, ^43^.

Based on previous studies, oxidative stress is one of the main mechanisms of CP‐induced nephrotoxicity.^44^ CP is a cytotoxic alkylation drug which needs to be activated by cytochrome P450 into its active metabolites that prevent cell division by inducing DNA cross‐linking.^45^ Acrolein is a CP metabolite which impairs the tissue antioxidant defense system. It stimulates the release of harmful reactive oxygen free radicals, and interacts with protein amino acids, leading to structural and functional changes in enzymes.^11^

In thisstudy, we investigated the effect of CP on MDA level as a marker of lipid peroxidation. CP treatment increased MDA levels in comparison to control group indicating CP‐induced oxidative stress. On the other hand, the renal MDA level was significantly reduced by co‐treatment with TOL as compared to CP group indicating that its nephroprotective effects may be based on its antioxidant properties. One of the limitations of our study was that we did not measure the other markers of the oxidative stress. However, we used the MDA as a key indicator for lipid peroxidation.

Our results are supported by a previous study where TOL activates the nuclear factor erythroid 2‐related factor 2/heme oxygenase (Nrf2/HO‐1) antioxidant pathway through phosphorylation of protein kinase RNA‐like endoplasmic reticulum kinase (PERK) in rodent models of chronic kidney disease.^46^

Several trials have verified the key role of NO in CP‐induced pathogenesis ^20^ and is involved in urothelial damage and inflammatory events, which leads to hemorrhagic cystitis.^47^ Where NO stimulates the production of pro‐inflammatory cytokines through the activation of NF‐κB in Kupffer cells.^48^ NF‐κB is a transcription factor that regulates the immune response and plays a central role in various inflammatory diseases in many organs.^49^

Our results revealed that, CP cumulative dose induced highly significant elevation in the tissue level of TNF‐α, NF‐κB, IL‐1β, and IL‐6 when compared to the control group.

Our study is going with previous studies which revealed activation of NF‐κB in response to oxidative stress, resulting in the stimulation of different inflammatory cytokines as TNF‐α, IL‐1β, and IL‐6, which cause tissue injury in CP‐challenged rats.^48^, ^50^

In our results, it was shown that CP‐induced apoptosis. We demonstrated the elevational level of apoptotic markers as caspase‐3 and Bax with decreased expression of antiapoptotic Bcl‐2 in renal tissue of CP group when compared to the control group. In agreement with our results, previous investigations revealed that CP could induce apoptosis in kidney tissue through increasing expression of apoptotic markers as caspase‐3 and Bax in renal tissue.^51^ Apoptosis is a programed cell death aiming to keep tissue homeostasis. Apoptosis is triggered by various stimuli including the oxidative damage resulting from the release of reactive oxygen species.^52^

Our results showed that, co‐treatment with TOL significantly reduced the pro‐inflammatory markers, TNF‐α, NF‐κB, IL‐1β, and IL‐6 in renal tissues as compared to CP‐treated group. Also, our results revealed antiapoptotic activity of TOL. Coadministration of TOL with CP induced significant decrease in the level apoptotic markers as caspase‐3 and Bax with significant increase in the expression of antiapoptotic Bcl‐2 in renal tissue as compared to CP‐treated group.

Ishikawa, Kobayashi, Sugiyama, Onoda and Ishimitsu, 2013 are in agreement with our study where they showed that TOL decrease the expression of both TNF‐α and NF‐κB in rat model of heart failure.^53^

Current finding could provide new interesting opportunity for developing new therapeutic approaches for nephroprotection against CP‐induced nephrotoxicity. TOL can correct CP‐induced hyponatremia and additionally it can provide protecting antioxidant, anti‐inflammatory, and antiapoptotic properties.

## CONFLICT OF INTEREST

Mohamed El‐Shabrawy, Amal Mishriki, Hisham Attia, Basma Emad Aboulhoda, Mohamed Emam, and Hanaa Wanas declare that they have no conflict of interest.

## ETHICAL APPROVAL

All the animal experiments were approved by the Institutional Animal Care and Use Committee of Cairo University, and were implemented in strict accordance with approved guidelines.

## Data Availability

Data available on request from the authors.
